# Identification of Urinary Biomarkers for Exercise-Induced Immunosuppression by iTRAQ Proteomics

**DOI:** 10.1155/2020/3030793

**Published:** 2020-01-23

**Authors:** Guoqin Xu, Wentao Lin, Andrew J McAinch, Xu Yan, Xiquan Weng

**Affiliations:** ^1^Department of Sports and Health, Guangzhou Sport University, Guangzhou 510500, China; ^2^Institute for Health and Sport, Victoria University, Melbourne 8001, Australia; ^3^Australian Institute for Musculoskeletal Science (AIMSS), Melbourne 3021, Australia

## Abstract

**Purpose:**

To identify noninvasive immune biomarkers of exercise-induced immunosuppression using the iTRAQ proteomics technique.

**Methods:**

Fifteen healthy males were recruited and subjected to a four-week incremental treadmill running training program. After each week of training, WBC counts and CD4^+^ and CD8^+^ lymphocytes were measured to monitor the immune function status. iTRAQ proteomics technology was used to identify differential proteins and their characteristics in urine.

**Results:**

Our data showed that the WBC counts, CD4^+^ lymphocytes, and CD4^+^/CD8^+^ ratio decreased by more than 10% after four weeks of training, suggesting exercise-induced immunosuppression. A total of 1854 proteins were identified in urine during the incremental running using the iTRAQ technology. Compared with the urine before training, there were 89, 52, 77, and 148 proteins significantly upregulated and 66, 27, 68, and 114 proteins significantly downregulated after each week, respectively. Among them, four upregulated proteins, SEMG-1, PIP, PDGFRL, and NDPK, increased their abundance with the increased exercise intensity. Bioinformatics analysis indicates that these proteins are involved in stress response and immune function.

**Conclusion:**

Four weeks of incremental treadmill running induced immunosuppression in healthy males. By using iTRAQ proteomics, four proteins in the urine, SEMG-1, PIP, PDGFRL, and NDPK, were found to increase incrementally with the increased exercise intensity, which have the potential to be used as noninvasive immune biomarkers of exercise-induced immunosuppression.

## 1. Introduction

Exercise-induced immunosuppression is a common medical problem that affects the training regimen in competitive sports [1]. During exercise-induced immunosuppression, the symptoms caused by acute respiratory infections may interfere with training and lead to the decline of mental attention, muscle strength, and aerobic ability during training for elite athletes [2, 3]. These symptoms significantly affect sports training and sports performance and increase athletes' risk of further illness and injury [1]. Although a large number of studies have shown that nutrient supplementation can effectively prevent the occurrence of exercise-induced immunosuppression [4–6], there are also studies suggesting that nutrient supplementation does not blunt the prolonged exercise-induced reduction in immunity [7]. Therefore, it is important to understand the characteristics of immune function with increased training load, which will play an important role in the early recognition and intervention of exercise-induced immunosuppression [8].

The degree of long-term exercise-induced impairment in the immune function of athletes mainly depends on exercise intensity [9, 10]. In 1902, Larrabee reported for the first time that the exercise load of a marathon exceeded the limits that the body could tolerate, as the body's inflammatory response such as phagocytosis was evident, and neutrophils increased significantly after a marathon race [11]. In the absence of food intake, long-term (>1.5 hours) moderate to high-intensity (50%–77% VO_2_max) exercise leads to the highest degree of immunological impairment [1]. In general, moderate exercise, defined as exercise intensity in the range of 40% to 60% of the maximum heart rate (HRmax) for 5 to 60 minutes, can enhance the body's immune function and reduce the incidence of upper respiratory tract infection (URTI) [12–14]. Excessive exercise, defined as 70% to 80% of HRmax for more than 60 minutes, has been demonstrated to have adverse effects on the immune system [15, 16]. As such, the infection rate of athletes who perform intense training increases significantly, indicating that once the training intensity reaches a threshold, the greater the intensity, the greater the immunosuppression and the risk of infection [17]. As a result, the relationship between exercise intensity/amount and URTI susceptibility forms a “J” curve [18–20], and the period of immunosuppression after high-intensity exercise is called “open window” period [18].

The early prediction of immune function changes by monitoring biomarkers in the course of high-intensity training or in the early stages of the competition to avoid exercise-induced immunosuppression is essential to optimize sports training and competition. Changes in immune response factors during intense training can be used as indicators of overtraining [21]. Kakanis et al. [22] found that, after 2 hours of 90% VO_2_ cycling exercise, CD4^+^ (Th1/Th2) cell levels changed immediately after exercise, while secreted cytokines such as IL-2, TNF (Th1), IL-6, and IL-10 (Th2) were altered 4 hours after exercise. Tuan et al. [23] conducted high-intensity exercise training for 3 consecutive days on healthy volunteers (VO_2_max 85%, 30 min per day) and found that the mitochondrial transmembrane potential (MTP) of peripheral blood leucocytes decreased immediately and was still lower 24 hours after the last exercise bout, which returned to normal level 72 hours after exercise. It is believed that the mitochondrial transmembrane potential is a functional marker of leukocyte viability and can be used to monitor the immune function of short-term high-intensity exercise. The above studies have shown that although many researchers try to find early diagnostic indicators of exercise-induced immunosuppression, they only focus on limited markers of immune function. The testing of immunomodulation indicators is complex and the monitoring of only a single biomarker and/or one time point often fails to provide a diagnosis [24]; thus, measuring the coexpression of multiple biomarkers and at multiple time points is essential for the diagnosis of immunomodulation [25].

Urine contains cellular and biochemical components derived from glomerular filtration of plasma, renal tubular excretion, and urogenital secretions [26]. Urinary proteins have been used as a noninvasive biomarker that can accurately monitor the body's stress under various psychophysiological changes and identify the body's condition in strenuous or unaccustomed exercise, competitions, overtraining, and improper recovery in sports [27, 28]. Urine is also an ideal source of biomarkers because it contains less lipid and more polypeptides, which are much higher in serum or tissue [29]. The development of proteomics technology provides a high-throughput, efficient, accurate, and sensitive research platform and enables the investigation of the relationship between exercise-induced immunomodulation and urine protein components [30].

In recent years, iTRAQ has been widely used as an *in vitro* labeling technique for polypeptides [31]. Extensive research [32–38] has shown that the application of iTRAQ technology can be used to identify disease-specific protein markers. Utilizing iTRAQ proteomics, this study aimed to determine the effects of 4 weeks of incremental treadmill running on the characteristics of urinary proteomics and then identify noninvasive immune biomarkers of exercise-induced immunosuppression.

## 2. Methods

### 2.1. Participants

Fifteen healthy males were recruited from the student cohort of Guangzhou Sport University. The inclusion criteria included the following: nonsmoker and nondrinker, no regular high-intensity exercise training, no medication two weeks before the experiment, and no severe exercise or fatigue in the past three days. The study was approved by the Guangzhou Sport University Ethics Committee. Subjects were informed of the aims and procedures of the study and provided written informed consent prior to enrolment into the study. The baseline characteristics of the subjects are shown in Table 1.

### 2.2. Exercise Protocol

**Figure 1:**
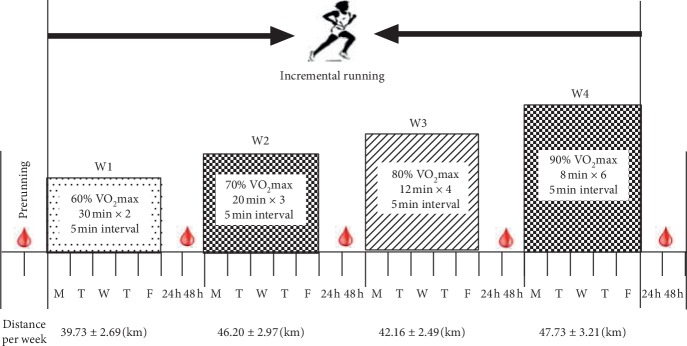
Four-week incremental treadmill running program and the distance per week.

All subjects underwent maximum oxygen uptake testing on a treadmill. The VO_2_max protocol began with an intensity of 8.0 km/h and consisted of increments of 1 km/h every 1 min by increasing the speed until volitional exhaustion. The criteria for reaching VO_2_max were determined by meeting at least 2 of the following [39]: (1) no further increase in oxygen uptake with increasing exercise workload, (2) a respiratory exchange ratio greater than 1.10, and (3) heart rate at or above the age-predicted maximal (using equation 220-age). The VO_2_max data was utilized to determine the corresponding running speed [40], and the subjects commenced 4-week incremental treadmill training 5 days per week, with training intensity adjusted each week [41]. Training intensity for the first week (W1) was 60% VO_2_max; for the second week (W2), 70% VO_2_max; for the third week (W3), 80% VO_2_max; and the fourth week (W4), 90% VO_2_max. The daily training program adopted an intermittent training mode, and the total running time was between 48 and 60 minutes. The detailed training program and the distance per week are shown in Figure 1.

### 2.3. Sample Collection

Before the training program, and at the end of each training week, fasting venous blood and midstream urine were collected at seven o'clock on each Sunday morning. Briefly, venous blood was collected using EDTA anticoagulant vacuum tubes to measure white blood cell count in the whole blood and CD4^+^ and CD8^+^ lymphocytes. The white blood cell count was measured within 4 h after blood was drawn [42], and the CD4^+^ and CD8^+^ lymphocytes were measured within 6 h after blood was drawn [43]. Following the collection of morning midstream urine, protease inhibitor cocktail (PMSF, 1 mmol/L, Amresco) was added to the urine samples to avoid proteolysis [44]. Urine samples were placed in liquid nitrogen and then stored at −80°C until further analysis.

### 2.4. Major Instruments and Reagents

The ADVIA® 120 Hematology System and Full Blood Count Test Kit were from Siemens, Germany. BD FACSCalibur, and FITC mouse anti-human CD3, APC mouse anti-human CD4^+^, and PE mouse anti-human CD8^+^, APC mouse IgG1, *ƙ* isotype control, PE mouse IgG1, *ƙ* isotype control, FITC mouse IgG1 *ƙ* isotype control, and CalibriteE 3-color kit were from BD Biosciences, USA. 8-plex iTRAQ Kit was from AB Sciex, USA. Pancreatin was from Promega, USA. First-dimension high pH-RP liquid chromatography was measured via Shimadzu HPLC, USA. Second-dimension liquid chromatography was measured via Thermo Dionex Ultimate 3000 RSLCnano, USA. Mass Spectrometer was performed on a Thermo Scientific Q Exactive, USA. The ELISA Test Kits of SEMG-1, PIP, PDGFRL, and NDPK were from Shanghai Enzyme-linked Biotechnology Co., Ltd., China. The Multiskan Spectrum was from Thermo Scientific, USA.

### 2.5. Whole Blood Leukocytes and Their Classification Tests

White blood cell count, neutrophil count, and lymphocyte count were measured using the ADVIA 120 hematology analyzer according to the manufacturer's instructions (Bayer Corporation, Germany).

### 2.6. Lymphocyte CD4^+^ and CD8^+^ Tests

Blood cells were analyzed on a FACScan (BD Biosciences, USA) equipped with CellQuest® software. The flow cytometer was calibrated and standardized with the CalibriteE 3-color kit before analysis (IVD, BD Biosciences, USA), and then FITC-CD3, PE-CD8, and APC-CD4 were analyzed by flow cytometry using standard protocols provided by the manufacturer.

### 2.7. Experimental Workflow of iTRAQ Labeling and Analysis

**Figure 2:**
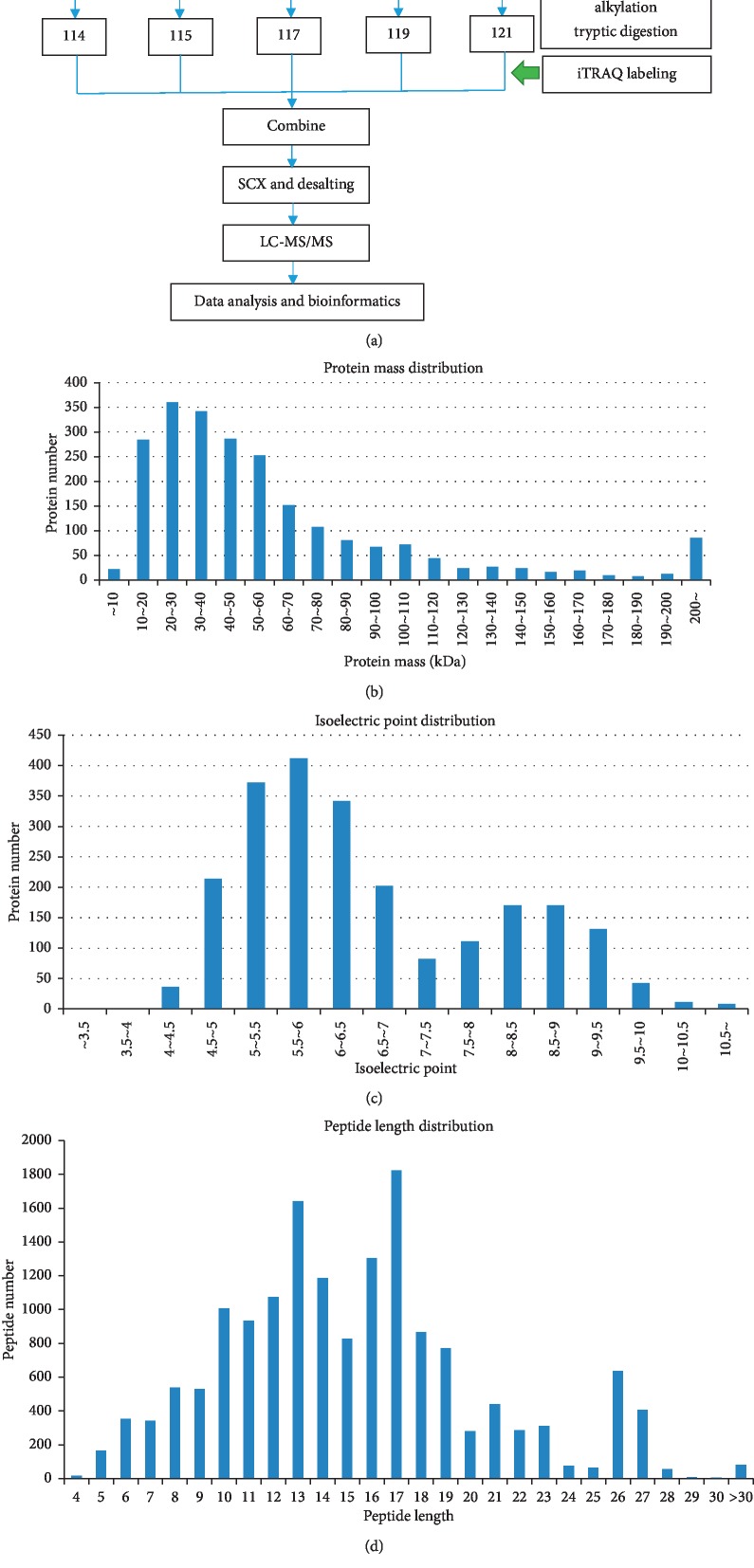
iTRAQ labeling and basic data analysis. (a) Experimental workflow for iTRAQ labeling and analysis. iTRAQ 8-plex labeling was performed on the collected urine samples. The labeled fractions were combined and subjected to strong cation exchange (SCX) chromatography and desalting, followed by separation using liquid chromatography mass spectrometry (LC-MS/MS), and data analysis and bioinformatics analysis. (b) Mass distribution of all the identified proteins. The horizontal axis is the molecular weight of the identified protein (Unit: kiloDalton, kDa). The vertical axis is the number of proteins identified. (c) The isoelectric point map of all the identified proteins. The horizontal axis is the isoelectric point of the identified protein and the vertical axis is the number of proteins identified. (d) Peptide length distribution of all the identified proteins. The graph shows the percentage of peptides of different lengths in all peptides. The abscissa is the number of peptide amino acid residues, and the ordinate is the number of peptides of this length.

In this study, the urine samples collected from all the participants at baseline and at the end of each week were thawed and combined into one urine sample for each time point and labeled using iTRAQ Reagent 8-plex kit (Sciex). The urine sample before the commencement of the training intervention (UW0) was labeled with labeling reagent 114, and the urine samples following the first week of training (UW1), the second week (UW2), the third week (UW3), and the fourth week (UW4) were labeled with labeling reagents 115, 117, 119, and 121, respectively. Each labeled urine sample was analyzed twice via iTRAQ, with two technical replicates of each sample [45]. The basic flow of the iTRAQ quantitative proteomics experiment [46] is shown in Figure 2.

### 2.8. Database Search and Screening for Differential Proteins

Protein searches were performed using the ProteinPilotTM Software 5.0 (AB Sciex). After the search was completed, the unused value was selected in the database Uniprot human, unused (ProtScore) for the search result (iTRAQ original data), unused ≥ 1.3 was set, and a protein with a confidence level of 95% or more was set as the analysis target. ProteinPilotTM FDR analysis was performed and the records beginning with “RRRRRR” in the search results were removed, and proteins with no quantitative information and proteins with poor repeatability (|*C*.*V*| ≥ 0.5) were removed. On the basis of protein analysis, differential proteins were screened. AVG ≥ 1.5 was set to indicate an upregulated protein, and AVG ≤ 0.67 was set to indicate a downregulated protein [47].

### 2.9. Bioinformatics Analysis

For the “co-upregulation” and “co-downregulation” of differential proteins in incremental treadmill running exercises, comprehensive biological functional annotations were performed using the Uniprot database website and DAVID data analysis software (http://david.abcc.ncifcrf.gov/home.jsp) [48]. To obtain the overall distribution of differentially expressed proteins in various functions, genetic enrichment analysis based on Gene Ontology (GO) of biological processes, cellular component, and molecular functions was performed, and the functional information of differentially expressed proteins was obtained. According to the biological functions of the differential proteins and the physiological reaction processes involved in the organism, GO clustering was performed to analyze the possible relationships between the differential proteins and the signal pathways that these proteins may participate in. The differential protein interaction network was analyzed by the string database [49, 50] (https://string-db.org/) to observe the interaction patterns between the differential proteins and to explore the relationship between them and the changes in immune function during the incremental treadmill running program.

### 2.10. ELISA Tests

The protein content of SEMG-1, PIP, PDGFRL, and NDPK was tested according to the manufacturer's instructions of the ELISA test kits (Shanghai Enzyme-linked Biotechnology, China), using a Multiskan Spectrum (Thermo Scientific, USA).

### 2.11. Statistical Analysis

Data was analyzed using IBM SPSS 22.0 software. One-way ANOVA was used for analyzing the related data. Data were expressed as mean ± standard deviation. *P* < 0.05 was considered statistically significant.

## 3. Results

### 3.1. Changes in Immune Function

#### 3.1.1. Changes in Leukocyte Counts in Blood

The white blood cell count showed a progressively decreasing trend and was 15% lower by the end of the 4th week (W4) of the incremental training, compared with that before training (W0) (Table 2; *P* < 0.05). The major reduction in leukocyte counts was neutrophils, which progressively decreased throughout the training program (W4 was 27.4% lower than that of W0, Table 2; *P* < 0.05). There was no significant difference in the lymphocyte count (Table 2).

#### 3.1.2. Changes of CD4^+^ and CD8^+^ Lymphocytes in Blood

The percentage of CD4^+^ lymphocytes was 11.8% lower by the end of the fourth week compared with W0 (Table 3; *P* < 0.05). Conversely, the percentage of CD8^+^ lymphocytes increased after 4 weeks of the incremental load training program (by 27.3%, *P* < 0.05). Of note, there were also significantly higher CD8^+^ lymphocytes at W1 (Table 3; *P* < 0.05) and W3 (Table 3; *P* < 0.05). The ratios of CD4^+^/CD8^+^ lymphocytes were significantly lower in W1, W2, W3, and W4 compared to W0 (Table 3; *P* < 0.05).

### 3.2. Differential Protein Status in Urine Proteomics during the Change in Immune Function

By performing proteomics quantitative measurements in the urine, it was found that there were differential proteins in the subject's combined urine samples after each one-week training period. As shown in Table 4, compared with preexercise urine (UW0), there were 87 upregulated and 64 downregulated proteins in the urine after the first week of exercise (UW1). After the second week of the exercise, the number of differential proteins in urine (UW2) was significantly reduced. Subsequently, the number of differential proteins in urine gradually increased with the increase in exercise intensity, especially for the number of differential proteins in urine after the 4th week of intense exercise (UW4). Compared with UW0, both the number of upregulated proteins and downregulated proteins were almost doubled.

**Figure 3:**
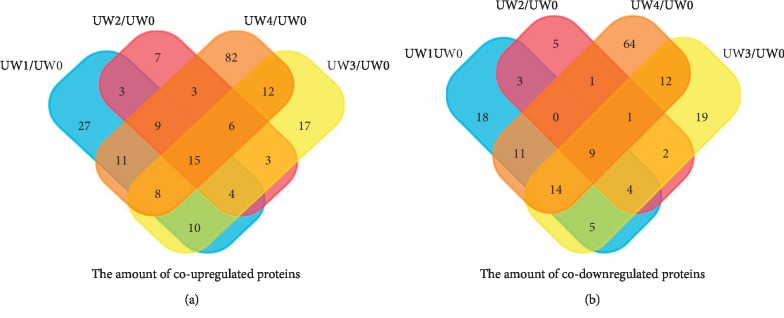
Compared with UW0, the amount of co-upregulated and co-downregulated proteins among the UW1, UW2, UW3, and UW4. (a) The amount of co-upregulated differential proteins. There were 15 co-upregulated proteins in the urine after each week of training compared to UW0. (b) The amount of co-downregulated differential proteins. There were 9 co-downregulated proteins in the urine after each week of training compared to UW0.

As shown in Figure 3 and Tables 5 and 6, further analysis of the differential proteins showed that there were 15 co-upregulated proteins and 9 co-downregulated proteins in the urine after each week of training compared to UW0. Among them, four upregulated proteins, including Semenogelin-1, Prolactin-inducible protein, Platelet-derived growth factor receptor-like protein, and Nucleoside diphosphate kinase, increased with increasing exercise intensity. In addition, some differential proteins increased with exercise intensity from the second week of the exercise, such as Glycerol-3-phosphate phosphatase, Secretogranin-1, Prosaposin, and Nephronectin (Fragment). Some proteins also decreased further with exercise intensity from the second week of the exercise, such as Immunoglobulin kappa constant, Immunoglobulin lambda variable 3–21, Signal peptide CUB and EGF-like domain-containing protein 2, and Uromodulin.

### 3.3. Bioinformatics Analysis

**Figure 4:**
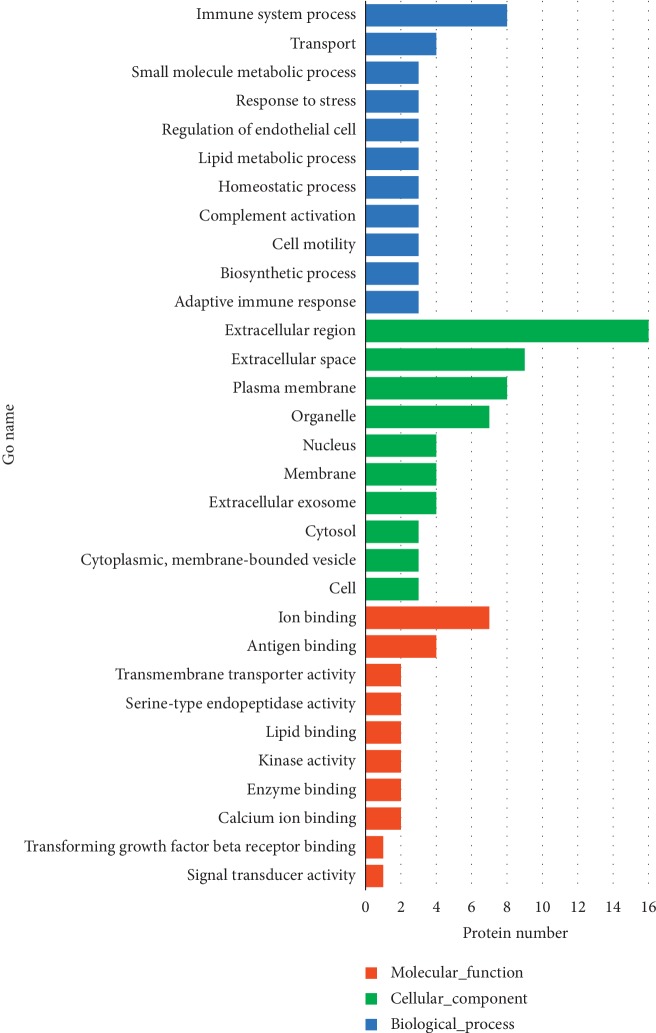
Gene Ontology (GO) annotation of 24 codifferential proteins. The codifferential proteins were divided into 3 categories: molecular function (MF), cellular component (CC), and biological process (BP). Each enumerated annotation is assigned by the enrichment protein number. The top 10 components for MF, CC, and BP of the codifferential proteins according to the GO database are shown.

To further understand the role of the above 24 differential proteins in immune function during the incremental treadmill-training program, we used the Uniprot database website and DAVID data analysis software to analyze the signaling pathways that may be related to these proteins and the possible differences between these proteins.  Figure 4 shows various GO enrichments of 24 differential proteins, including biological processes (top three: immune system processes, transport, and small molecule metabolic process), molecular functions (top three: ion binding, antigen binding, and transmembrane transporter activity), and cellular composition (top three: extracellular area, extracellular space, and plasma membrane).

### 3.4. Transcriptional Regulatory Network between Differential Proteins

**Figure 5:**
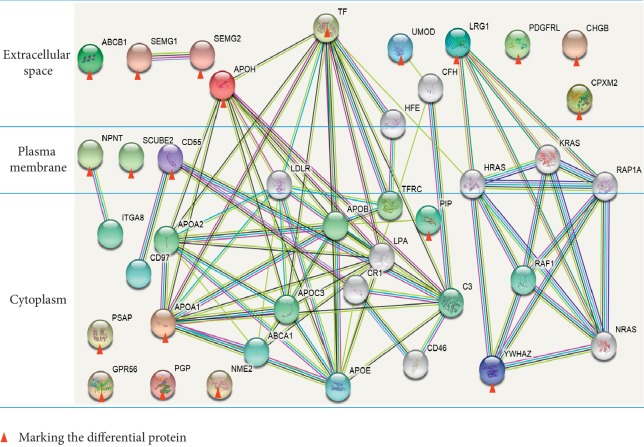
The genes of differential proteins in the interaction network. For functional enrichment in the network, biological process (GO), in the pathway analysis, there are 10 genes count in gene set pathways of positive regulation of response to stimulus (pathway ID, GO: 0048584). Molecular Function (GO), there are two genes count in gene set pathways of IgG binding (pathway ID, GO: 0019864). The red line shows Gene Fusions, the green line indicates Neighborhood in the Genome, the blue line indicates Cooccurrence across Genomes, the purple line indicates Experimental/Biochemical Data, the yellow line indicates text mining evidence, the light blue line indicates database evidence, and the black line indicates coexpression evidence.

The analysis of the 24 differential proteins interaction networks through the STRING database (https://string-db.org/) was undertaken. No corresponding genes were found for four proteins by the name in Homo sapiens, including IGKC, IGLV3-21, IGLV4-69, and IGLV2-18, and the genes of the other 20 proteins in the interaction network are shown in Figure 5. For functional enrichment in the network, biological process (GO), in the pathway analysis, there are 10 genes count in gene set pathways of positive regulation of response to stimulus (pathway ID, GO: 0048584). This pathway also includes ten differential proteins, namely, Apolipoprotein A-I (APOA1), Beta-2-glycoprotein 1 (APOH), Complement decay-accelerating factor (CD55), Adhesion G-protein-coupled receptor G1 (Fragment) (GPR56), Leucine-rich alpha-2-glycoprotein (LRG1), Nucleoside diphosphate kinase (NME2), Nephronectin (Fragment) (NPNT), Prosaposin (PSAP), Serotransferrin (TF), and 14-3-3 protein zeta/delta (Fragment) (YWHAZ). For molecular function (GO), there are two genes count in gene set pathways of IgG binding (pathway ID, GO: 0019864). This pathway also contains two differential proteins, Prolactin-inducible protein (PIP), and Uromodulin (UMOD). This pathway is associated with the immunological function of the organism together with three other proteins: Immunoglobulin lambda variable 3–21, Immunoglobulin lambda variable 4–69, and Immunoglobulin lambda variable 2–18.

### 3.5. ELISA Validation

Four of the identified differential proteins in the urine were validated by ELISA (Table 7). During incremental running, the content of SEMG-1, PIP, PDGFRL, and NDPK was increased with increasing exercise intensity. Compared with W0 (before training), upregulation was observed for PIP and NDPK from W1, SEMG-1 from W2, and PDGFRL from W3 (Table 7; *P* < 0.05).

## 4. Discussion

In the current study, we found that WBC counts, CD4^+^ lymphocytes, and CD4^+^/CD8^+^ ratio decreased after four weeks of incremental treadmill training, suggesting exercise-induced immunosuppression. By using iTRAQ technology, we have identified a total of 1854 proteins in the urine samples. Compared with the urine before training, there were 89, 52, 77, and 148 proteins significantly upregulated and 66, 27, 68, and 114 proteins significantly downregulated after each week, respectively. Among them, four upregulated proteins, SEMG-1, PIP, PDGFRL, and NDPK, increased their abundance with the increased exercise intensity. We further used bioinformatics analysis and found that these proteins are involved in stress response and immune function. To our knowledge, this is the first study to employ the iTRAQ technology to analyze urine proteomics of exercise-induced immunosuppression. Our data suggested that some urine proteins could be used as biomarkers of exercise-induced immunosuppression, which are relatively easy to monitor athletes.

High-intensity and large-volume exercise training can cause significant changes in the immune parameters of the body's nonspecific and specific immune system, especially the response of NK cells, and neutrophils, leading to the body's immunosuppression during a heavy training workload and for a few days after the training [18]. It has been widely reported that long-term high-intensity exercise training will have a strong negative impact on the immune function of the athletes, resulting in inhibition of immune function, increased susceptibility to various infectious diseases, and even lead to poor performance during intensive training [51]. This study confirmed that the subjects' white blood cell count, neutrophil count, CD4^+^, and CD4^+^/CD8^+^ ratio were decreased significantly after 4 weeks of incremental treadmill exercise. Although the exercise intensity was relatively small during the first two weeks, the accumulation of daily exercise training also reduced the immune function. In particular, following the fourth week of high-intensity exercise training, a significant decrease was observed in the body's immune function (immunosuppression). This was evidenced by reductions in white blood cell count (15%), neutrophil count (27.4%), CD4^+^ lymphocytes (11.8%), CD4^+^/CD8^+^ ratio (34.6%), and an increase in CD8^+^ lymphocytes (27.3%). The most important clinically relevant finding during exercise-induced immunosuppression is a higher risk of URTI [52]. While reported rates for URTI vary, up to 50% of fitness enthusiasts in high school and university training rooms have experienced respiratory infections [15]. It has also been reported that the occurrence rate of URTI for competitive swimmers exceeded 40% after 4 weeks of intense training [53], while 12 weeks of high-intensity training increased the incidence of URTI in tennis players [54].

It is interesting to note that the decline in the immune system can be accompanied by the contemporary occurrence of proteinuria during long and strenuous exercise [55]. The reason for the large number of differential proteins in the urine collected following the first week of training may be due to the subject's inability to adapt to the consecutive training of the first week [56]. As the body slowly adapts during the second week, the number of differential proteins in the urine is significantly reduced. When the exercise load increases, the number of differential proteins in the urine gradually increases, especially with high-intensity exercise. It has been shown previously that intense exercise-induced proteinuria is related to exercise intensity [57, 58] and that the doubling of urine proteins caused by high-intensity exercise in the fourth week suggests an increased glomerular permeability and kidney secretion caused by exercise stress. It has been previously reported that exercise intensity affects kidney function more than exercise load [59].

Of all the differentially expressed urinary proteins induced by incremental training, 15 proteins are consistently upregulated in all four time points, and 9 proteins are consistently downregulated in all four time points. Among them, four proteins SEMG-1, PIP, PDGFRL, and NDPK increased their abundance with increasing exercise intensity. SEMG-1 has been detected in the gastrointestinal tract, skeletal muscle [60], and normal human urine [61]. It is processed and presented by thymic antigen presenting cells and is likely to participate in shaping the T-cell repertoire [62], which is involved in the regulation of immune function [63, 64]. SEMG-1 is upregulated in exosomes secreted by vascular endothelial cells under hypoxic stress conditions [65] and is considered as a new predictor of renal damage [66]. PIP also plays an important role in immune regulation. It is abundantly present in saliva and bronchial submucosal glands and exerts mucosal immunity [67]. PIP binds to immunoglobulin G (IgG) [68] and CD4-T cell receptor [69, 70] to exert important biological functions [71–75]. The expression of PIP is regulated by immunoregulatory hormones, such as androgen and glucocorticoids [76]. Lack of PIP leads to impaired Th1 immune response [77]. The expression of PDGFRL is associated with blood pressure, heart quality, and insulin sensitivity [78, 79], while NDPK regulates angiogenesis [80], neuronal protection [81], and insulin secretion regulation [82]. Taken together, previous studies suggested that the differential protein abundance of SEMG-1, PIP, PDGFRL, and NDPK in urine is related to the immune function and stress response. Our current results show that while the body's immune function is declining, as evidenced by the decreased white blood cells, CD4^+^ and CD4^+^/CD8^+^ ratio, the four upregulated proteins in urine gradually increase during the incremental treadmill-training program. We, therefore, conclude that SEMG-1, PIP, PDGFRL, and NDPK may serve as noninvasive immune biomarkers for exercise-induced immunosuppression.

Through bioinformatics analysis, it was found that, during the process of exercise-induced immunosuppression, the components of the differential proteins in urine are mainly distributed in the extracellular region, extracellular space, and plasma membrane. The reason may be due to the increased destruction of the plasma membrane by free radicals, and proteins in the extracellular area, extracellular space, and the plasma membrane are more likely to enter the bloodstream [83] and then are filtered out with the increased permeability of the glomerular filtration membrane into the urine [84, 85]. The biological processes of these differential proteins mainly perform immune system processes, transport, and small molecule metabolic processes.

Further analysis of the functional enrichments of these differential protein-gene interaction networks revealed that 10 genes of the differential proteins are related to the pathway of positive regulation of response to the stimulus in the biological process. In molecular function, 5 genes of the differential proteins are in the pathway of IgG binding. It is likely that the incremental treadmill running mobilized the body's intense stress response and adaptive immune compensation [86]. Meanwhile, due to the increase of free radicals and the relative lack of free radical scavenging ability induced by exercise stress [87], the disruption of normal membranous structures and the increase of cell membrane permeability initiated. As the ultrastructural changes of the glomeruli and tubules and the permeability of glomerular filtration membranes increase, protein filtration rate increases and the secretion of proteins by the kidney increases [88], resulting in an increased presence of urinary proteins, such as those related to stress regulation and immune function. Therefore, testing these proteins excreted in the urine can indirectly reflect the body's stress and immune function inhibition.

In summary, this current study has identified four differential proteins, SEMG-1, PIP, PDGFRL, and NDPK, as noninvasive immune biomarkers in urine for exercise-induced immunosuppression. Further studies are needed to validate the noninvasive biomarkers of exercise-induced immunosuppression, possibly in a different population (especially in females or elite athletes) or with a different training program. The sensitivity and specificity of each biomarker also need to be tested in future studies.

## Figures and Tables

**Table 1:** Subject characteristics.

Age (y)	Height (cm)	Weight (kg)	BMI (kg/m^2^)	RPE	Relative VO_2_max (ml/kg/min)	HRmax (bpm)
19.2 ± 0.7	170.6 ± 4.9	61.55 ± 6.58	20.70 ± 1.41	6.1 ± 0.4	48.60 ± 4.98	200.7 ± 0.8

BMI: body mass index; RPE: ratings of perceived exertion.

**Table 2:** Changes of white blood cell counts and their classification counts during 4 weeks of incremental treadmill running.

	W0	W1	W2	W3	W4
White blood cell count (×109)	7.03 ± 1.70	6.56 ± 1.34	6.25 ± 1.06	6.49 ± 0.94	5.97 ± 0.95^*∗*^
Neutrophil count (×109)	3.71 ± 1.61	3.22 ± 0.96	3.07 ± 0.68	3.16 ± 0.69	2.69 ± 0.60^*∗∗*^
Lymphocyte count (×109)	2.57 ± 0.50	2.61 ± 0.58	2.48 ± 0.37	2.58 ± 0.44	2.51 ± 0.64

^*∗*^ *p* < 0.05; ^*∗∗*^*p* < 0.01, compared with W0.

**Table 3:** Changes of CD4^+^ and CD8^+^ lymphocytes in the blood during 4 weeks of incremental treadmill running.

	W0	W1	W2	W3	W4
CD4+ (%)	54.66 ± 7.84	51.36 ± 6.26	50.90 ± 7.99	51.14 ± 5.25	48.19 ± 5.61^*∗∗*^
CD8+ (%)	32.23 ± 9.04	39.77 ± 7.81^*∗∗*^	36.97 ± 8.46	38.26 ± 4.24^*∗*^	41.03 ± 7.07^*∗∗*^
CD4^+^/CD8^+^	1.88 ± 0.73	1.37 ± 0.47^*∗∗*^	1.50 ± 0.58^*∗*^	1.36 ± 0.25^*∗∗*^	1.23 ± 0.36^*∗∗*^

^*∗*^ *p* < 0.05; ^*∗∗*^*p* < 0.01, compared with W0.

**Table 4:** Changes in the number of differential proteins in urine during exercise-induced immunosuppression.

	Compared with UW0		Compared with UW1		Compared with UW2		Compared with UW3	
	Upregulation	Downregulation	Upregulation	Downregulation	Upregulation	Downregulation	Upregulation	Downregulation
UW1	87	64						
UW2	50	25	36	35				
UW3	75	66	44	52	42	42		
UW4	146	112	101	108	113	115	104	90

**Table 5:** Multiples of differential co-upregulation protein content in urine during exercise-induced immunosuppression.

Accession	Name	Gene	UW1/UW0	UW2/UW0	UW3/UW0	UW4/UW0
A6NDG6	Glycerol-3-phosphate phosphatase	PGP	6.402	1.579	5.065	6.770
P02647	Apolipoprotein A-I	APOA1	2.838	2.036	5.556	1.616
P02787	Serotransferrin	TF	1.641	2.126	3.919	1.542
P04279	Semenogelin-1	SEMG1	5.971	6.339	32.16	38.385
P05060	Secretogranin-1	CHGB	2.981	2.452	2.907	6.679
P08183	Multidrug resistance protein 1	ABCB1	2.754	3.133	1.888	1.941
P12273	Prolactin-inducible protein	PIP	51.665	50.957	56.903	61.536
Q02383	Semenogelin-2	SEMG2	7.977	7.670	28.796	40.938
Q15198	Platelet-derived growth factor receptor-like protein	PDGFRL	2.489	3.311	21.878	22.699
Q8N436	Inactive carboxypeptidase-like protein X2	CPXM2	1.791	1.850	1.683	1.583
C9JIZ6	Prosaposin	PSAP	2.382	1.863	4.002	8.306
D6RH31	Nephronectin	NPNT	1.969	1.611	1.739	2.605
E7EX29	14-3-3 protein zeta/delta	YWHAZ	1.786	1.675	1.556	2.512
H3BSN7	Adhesion G-protein-coupled receptor G1	GPR56	5.248	4.831	4.875	4.131
Q32Q12	Nucleoside diphosphate kinase	NME2	1.905	1.905	1.585	2.188

**Table 6:** Multiples of differential co-downregulation protein content in urine during exercise-induced immunosuppression.

Accession	Name	Gene	UW1/UW0	UW2/UW0	UW3/UW0	UW4/UW0
P01834	Immunoglobulin kappa constant	IGKC	0.308	0.667	0.530	0.113
P02749	Beta-2-glycoprotein 1	APOH	0.615	0.605	0.619	0.556
P02750	Leucine-rich alpha-2-glycoprotein	LRG1	0.264	0.400	0.606	0.443
P80748	Immunoglobulin lambda variable 3–21	IGLV3-21	0.520	0.603	0.337	0.211
A0A075B6H9	Immunoglobulin lambda variable 4–69	IGLV4-69	0.501	0.441	0.619	0.437
A0A075B6J9	Immunoglobulin lambda variable 2–18	IGLV2-18	0.643	0.592	0.608	0.570
A0A0A0MTC8	Signal peptide, CUB and EGF-like domain-containing protein 2	SCUBE2	0.545	0.667	0.643	0.550
H7BY55	Complement decay-accelerating factor	CD55	0.637	0.637	0.347	0.608
X6RBG4	Uromodulin	UMOD	0.453	0.631	0.477	0.349

**Table 7:** Changes of differential proteins in the urine during 4 weeks of incremental treadmill running.

	W0	W1	W2	W3	W4
SEMG1 (*μ*g/ml)	2.57 ± 0.81	3.63 ± 1.26	4.13 ± 1.40^*∗∗*^	4.51 ± 1.72^*∗∗*^	6.61 ± 2.35^*∗∗*^
PIP (*μ*g/ml)	55.68 ± 18.38	79.98 ± 24.85^*∗*^	81.45 ± 21.97^*∗*^	84.55 ± 29.31^*∗∗*^	113.79 ± 37.32^*∗∗*^
PDGFRL (*μ*g/ml)	4.29 ± 1.01	5.61 ± 2.08	5.64 ± 2.12	7.30 ± 1.92^*∗∗*^	10.98 ± 3.38^*∗∗*^
NDPK (*μ*g/ml)	9.82 ± 3.20	15.70 ± 4.54^*∗*^	16.54 ± 5.37^*∗∗*^	20.39 ± 8.54^*∗∗*^	29.91 ± 8.37^*∗∗*^

^*∗*^ *p* < 0.05, ^*∗∗*^*p* < 0.01, Compared with W0.

## Data Availability

The ZIP data used to support the findings of this study are available from the corresponding author upon request.
